# Mitophagy in Parkinson’s Disease: Pathogenic and Therapeutic Implications

**DOI:** 10.3389/fneur.2017.00527

**Published:** 2017-10-04

**Authors:** Fei Gao, Jia Yang, Dongdong Wang, Chao Li, Yi Fu, Huaishan Wang, Wei He, Jianmin Zhang

**Affiliations:** ^1^Department of Immunology, Research Center on Pediatric Development and Diseases, Institute of Basic Medical Sciences, Chinese Academy of Medical Sciences and School of Basic Medicine, Peking Union Medical College, State Key Laboratory of Medical Molecular Biology, Beijing, China

**Keywords:** mitophagy, Parkinson disease, mitochondria, synuclein, autophagosome

## Abstract

Neurons affected in Parkinson’s disease (PD) experience mitochondrial dysfunction and bioenergetic deficits that occur early and promote the disease-related α-synucleinopathy. Emerging findings suggest that the autophagy-lysosome pathway, which removes damaged mitochondria (mitophagy), is also compromised in PD and results in the accumulation of dysfunctional mitochondria. Studies using genetic-modulated or toxin-induced animal and cellular models as well as postmortem human tissue indicate that impaired mitophagy might be a critical factor in the pathogenesis of synaptic dysfunction and the aggregation of misfolded proteins, which in turn impairs mitochondrial homeostasis. Interventions that stimulate mitophagy to maintain mitochondrial health might, therefore, be used as an approach to delay the neurodegenerative processes in PD.

## Introduction

Parkinson’s disease (PD) is an incurable chronic progressive disease affecting nearly 2% of the “over 50” population with an approximately estimate of more than 6 million cases worldwide (1). The cause of PD is generally unknown, but it is believed to involve both genetic and environmental factors (2). Epidemiological studies have revealed that fewer than 10% of PD cases are inherited from family, whereas the majority of cases are sporadic (3). Discoveries of genes linked to rare familial forms of PD have confirmed the critical role of genes in the development of PD and made great contributions in understanding the molecular pathogenesis behind this common but complex illness. Autophagy is a conserved pathway that degrades damaged organelles and misfolded proteins (4). Here, we consider the roles of autophagy in neuronal health and the pathological mechanisms leading to disease progression to help us seek for potential targets for neuroprotective interventions, which may revolutionize the treatment of this incurable disease.

## Protein Aggregation and Mitochondrial Dysfunction in PD

Parkinson’s disease is a neurodegenerative movement disorder characterized by the preferential loss of dopaminergic neurons in the substantia nigra, which results in progressive motor system malfunction (5). Primary motor signs that characterize PD include rigidity, bradykinesia, postural instability, and tremor (6). The pathology of PD remains unknown, but almost all cases show the presence of intraneuronal misfolded protein aggregates forming Lewy bodies, the primary component of which is α-synuclein (7). Protein homeostasis is crucial to sustain cellular health and viability in neurons (8). The process of α-synuclein accumulation resulting in the generation of highly diffusible small oligomers and fibrils, which abnormally aggregate and can be visualized as eosinophilic cytoplasmic inclusion in neurons (9). Evidence indicates that the accumulation of [α-synuclein, possibly oligomers, without insoluble aggregates, may lead to oxidative stress and give rise to deleterious effects in dopamine (DA) neurons (10–13)].

Recent evidence suggests that α-synuclein is a lipophilic protein, localized to mitochondria and connected to endoplasmic reticulum (ER) through mitochondrial-associated ER membrane (MAM) (14, 15). Overexpression of α-synuclein inhibits the normal function of inner-mitochondrial membrane-anchored respiratory chain complexes in whole brain of PD patients, but mostly in nigrostriatal neurons. Increased levels of reactive oxygen species (ROS) might be the cause of neuronal death (16). A study has also demonstrated that α-synuclein overexpression in mitochondria increases the number of fragmented mitochondria in vitro (17). In addition, intermediate α-synuclein accumulation (pre-fibrillar forms) reduces mitochondrial Ca^2+^ retention (18). Ca^2+^ is required by mitochondria for the generation of ATP *via* the tricarboxylic acid cycle (19). Perturbed neuronal Ca^2+^ levels caused by soluble pre-fibrillar α-synuclein lead to altered mitochondrial membrane potential and NADH oxidation, which indicate the dysfunction of complex I (20). The effect of complex I inhibitor 1-methyl-4-phenyl-1,2,3,6-tetrahydropyridine (MPTP) and its active metabolite 1-methyl-4-phenylpyridinium (MPP+) on dopaminergic cell death is inhibited in mouse models lacking α-synuclein, which is mainly due to the inactivation of nitric oxide synthase (NOS) (21). In addition, siRNA-mediated knockdown of α-synuclein also protects cells from NOS activation in cellular models, rescuing cells from MPP^+^-induced apoptosis (22).

Posttranslational modification of α-synuclein is also a crucial factor in the pathological mechanisms of PD. Many PD-associated mutations in α-synuclein also induce mitochondrial dysfunction. The H50Q mutation is proved to induce aggregation of α-synuclein oligomers in SH-SY5Y cells and increase the number of fragmented mitochondria in hippocampal neurons *in vivo* (23, 24). Ser129-induced α-synuclein aggregation is involved in the formation of Lewy bodies and plays a critical role in the neurodegenerative process (25). SH-SY5Y cells expressing A53T α-synuclein exhibit depolarized mitochondrial and increased ROS levels when exposed to rotenone (26). Studies in transgenic mice overexpressing the A53T-mutant human α-synuclein revealed that intracerebral inoculation of aggregated α-synuclein or preformed recombinant α-synuclein fibrils induces a progressive and ultimately lethal α-synucleinopathy in inoculated animals (27, 28).

Damaged cellular function and decreased ATP levels induced by α-synuclein are detrimental to dopaminergic neurons and provide implications for disease pathogenesis in PD. Impaired mitochondrial function may lead to a reduction in cellular energy levels and excessive ROS production in neurons, which in turn exacerbate mitochondrial damage (29). As a result, measures to enhance the degradation of abnormally aggregated proteins and the clearance of damaged mitochondria seem to be the most promising strategies in rescuing neurodegeneration in PD patients.

## PD-Related Genes and Their Roles in Mitophagy and Mitochondrial Dysfunction

Autophagy is an evolutionarily conserved process in which cytoplasmic substrates are engulfed in autophagic vesicles and fused to lysosomes for degradation and recycling (30). The specific autophagic elimination of mitochondria is defined as mitophagy (31). Autophagy is classified into various subgroups based on the mechanism of substrate delivery to the lysosome, including macroautophagy, chaperone-mediated autophagy (CMA), and microautophagy (4). The process of mitophagy is directed mainly by macroautophagy. Genome-wide association studies implicate that PD-related genes and their products are responsible for mitochondrial homeostasis and mitophagy (32, 33).

PINK1 and Parkin are the most well-known proteins related to PD. PINK1, encoded by PARK6 gene, is a mitochondrial-targeted serine/threonine kinase, while Parkin, encoded by the PARK2 gene, is a 465-amino acid E3 ubiquitin ligase (34, 35). “Loss-of-function” mutations in either PINK1 or Parkin lead to autosomal recessive forms of PD (35, 36). PINK1-dependent activation of Parkin is recognized as a major pathway of mitophagy (37). When mitochondria become depolarized, PINK1 accumulates on the surface of the outer membrane of mitochondria, where it phosphorylates both ubiquitin and Parkin and activates Parkin’s ubiquitin E3-ligase activity. Moreover, it was recently shown that wild-type PINK1 recruits Parkin to damaged mitochondria during mitophagy rather than the PD-linked PINK1-mutant forms (38). The subsequent recruitment of ubiquitin-binding mitophagy receptors lead to the formation of LC3-positive phagophores, which sequester damaged mitochondria from the cytosol and eventually degrade by lysosomal hydrolases (39). PINK1 and Parkin are also important for sustaining mitochondrial homeostasis through the regulation of mitochondrial fission and fusion. A study has shown that the ubiquitination process of mitochondrial fusion protein mitofusin (Mfn) is mediated by both PINK1 and Parkin. Loss of PINK1 or Parkin causes damaged mitophagy process and elongated mitochondria in *Drosophila* (40). Genetic loss of Mfn1 and Mfn2 leads to the dissipation of membrane potential in a subset of mitochondria, preventing Parkin’s recruitment process through the translocase of the inner membrane complex (41). Parkin-mutant or PINK1-mutant *Drosophila* display a severe defect in flight muscle, leading to behavioral locomotive problems and greater susceptibility to oxidative stress (42, 43). Indirect flight muscles and DA neurons in this model are filled with swollen mitochondria (44, 45). Mutant-Parkin displays degeneration of a subset of DA neurons, exhibiting shrinkage in morphology and decreased DA level in *Drosophila* brains (42, 46, 47). PINK1 knockout fibroblasts and neurons exhibit reduced membrane potential, overloaded Ca^2+^ levels and increased ROS production in mitochondria (48, 49). Meanwhile, mitochondria isolated from the brain of PINK1 knockout mice show defects in Ca^2+^-buffering capacity and increased vulnerability of neurons in oxidative stress caused by inflammation (50). DA neuronal death is also observed in a conditional Parkin ablation mouse model after lentivirus delivers the Cre recombinase to the mouse brain, which suggests that Parkin plays an important role in neuronal survival (51).

Mutations in the PARK7 gene, which encode DJ-1, cause a rare autosomal recessive form of PD (52, 53). DJ-1, a transcriptional regulator, is often known as a redox sensor/reductase which influences mitochondrial homeostasis and mitophagy (54). It is long believed that DJ-1 is a neuroprotective factor (55). Mitochondria localized DJ-1 is a component of thioredoxin/apoptosis signal-regulating kinase 1 (Trx/Ask1) complex, which regulates the clearance of endogenous ROS through the modulation of scavenging systems (56). DJ-1 deficiency decreases brain mitochondria consumption of H_2_O_2_, leading to the increased level of oxidative stress, and eventually causes cell death in DA neurons (54, 57). In addition, DJ-1 directly interacts with α-synuclein. The mutant form of DJ-1 in PD causes misfolded α-synuclein aggregate in DA neurons, while the overexpression of DJ-1 reduces the dimerization of α-synuclein (55).

LRRK2 is a member of the leucine-rich repeat kinase family that is encoded by the PARK8 gene (58). Mutations in LRRK2 are associated with autosomal-dominant PD (33). Expression of mutant LRRK2 may have a variety of negative effects on mitochondrial and cellular health (59, 60). Endogenous LRRK2 directly interacts with the mitochondrial fission and fusion regulators dynamin-related protein 1, Mfn, and optic atrophy 1 (OPA1) to maintain the balance among mitochondrial biogenesis, intracellular material trafficking, metabolic demands, and mitochondrial morphology (61–63). G2019S mutant LRRK2 in sporadic PD patients showed decreased levels of OPA1, indicating that LRRK2 kinase activity is also an important factor in mitochondrial dynamics (64). The overexpression of G2019S mutant LRRK2 in mouse brains showed mitochondrial uncoupling accompanying with an increased basal oxygen consumption in both fibroblast and neuroblastoma cells, resulting in decreased ATP level and compromised cellular function (65). Fibroblasts with G2019S mutant LRRK2 from PD patients showed increased susceptibility to MPP^+^ induced cell death (66). Meanwhile, the depletion of LRRK2 or mutant LRRK2 impair the autophagy/lysosomal pathway, leading to the accumulation of autophagosomes (67, 68). The degradation of LRRK2 in lysosomes is mediated by CMA in nervous system, while the mutant forms of LRRK2 and also high concentrations of wild-type LRRK2 interfere with the CMA translocation complex, resulting in defective CMA (67, 69). Inhibition of CMA in neurons induces the accumulation of both soluble and insoluble α-synuclein, which in turn could compromise the degradation of α-synuclein and initiate protein aggregation in PD (70, 71).

Lysosomal defects in the clearance of cytosolic substrates also contribute to the progression of PD (72). PARK9 encoded lysosomal ATPase ATP13A2 is a P-type transport ATPase which protects against cellular dysfunction caused by α-synuclein (73). PD-linked mutations in ATP13A2 reduce the activity of proteolytic processing enzymes, disturbing the acid environment in lysosomes, resulting in the impaired degrading capacity of autophagosomes (74).

As we can see, these PD-related genes not only play a role in the maintenance of mitochondrial homeostasis but also are important for the clearance of aggregated proteins and damaged organelles through mitophagy. Mitochondrial deficiency is responsible for neurodegeneration in PD, but the specific mechanism between mitochondrial deficiency and α-synuclein aggregation remains to be discovered.

## Therapeutic Implications for Pharmacological Targeting and Gene Therapy

Intracellular misfolded proteins contribute to cellular dysfunction and neuronal death in PD patients. Moreover, compromised clearance pathways aggravate the pathological process of this neurodegenerative disease. Since autophagy plays an important role in selectively degrading misfolded proteins and damaged organelles, it could be an interesting target for the development of efficient treatment for PD. Nowadays, up-to-date researches also give us implications on PD-related genes and their influence on mitochondrial homeostasis. The obstacles between this promising therapeutic targets and mitochondrial dynamic are still a major challenge for us to overcome.

Methods identified to enhance autophagy in several preclinical PD models are proven to be effective. The serine/threonine protein kinase mTOR is a component of the mTOR complex 1 and suppresses autophagy under nutrient-rich conditions (75). The mTOR inhibitor rapamycin, which stabilizes the association of mTOR complex and inhibits the kinase activity, is the most widely used small molecule drug which is proved effective in enhancing autophagy activity in many disease models (76–81). Rapamycin selectively suppresses the activity of mTOR through the dephosphorylation of Akt kinase, which is crucial for neuronal survival in PD models (82, 83).

Beclin 1 is encoded by autophagy-related gene 6. This protein interacts with either BCL-2 or the class III phosphatidylinositol 3-kinase (PI_3_K) VPS34, playing a critical role in the localization of other autophagy-regulatory proteins to the preautophagosomal structure (84). Beclin 1 is negatively regulated by BCL-2 and BCL-X_L_ at ER membranes (85). Mutations in BH3-related domain in Beclin 1 disrupt the formation of Beclin 1–BCL-2 complex, leading to enhanced autophagy (86). Chronic administration of trehalose results in a reduction of the frontal cortex p62/beclin 1 level, suggesting an elevated state of autophagy (87–90). Moreover, ER stress is responsible for the activation of autophagy through the unfolded-protein response (UPR) (91). Tunicamycin Induced mild ER stress shows a promising treatment potential in protecting dopaminergic neurons from death in PD models (92). Gene therapy approaches to handle the unfolded protein load *via* the activation of UPR are designed to manipulate autophagy in a more specific manner (93). Beclin 1 gene therapy mediated by lentivirus exhibits not only positive effects in the clearance of intraneuronal α-synuclein proteins but also a proved synaptic function in PD models (94). Gene therapy also exhibits great potential in the clearance of abnormally aggregated proteins in other neurodegenerative diseases through the activation of autophagy (95–97).

Although methods to activate autophagy are promising novel therapeutic approaches for PD, a complex scenario is emerging in which the alteration of distinct regulatory steps in autophagy may perturb the homeostasis of the cell, contributing to the disease progression as well (98). Therefore, the mere enhancement of autophagy may have detrimental consequences by provoking neurodegeneration and exacerbating disease progression. Thus, it is critical that this biological process should be precisely regulated and strictly monitored. Moreover, the specific mechanism behind each subtype of the disease that may link the defects of autophagy to PD still remains to be discovered. Considering the complex nature of PD, individualized interventional targets seem to be the most promising method for deciding the right timing and appropriate degree of activation of autophagy.

## Concluding Remarks

Significant progress has been made in understanding the causes of this neurodegenerative disorder. The accumulation of dysfunctional mitochondria and compromised mitophagy have emerged as common features of affected neurons in patients and animal models that may cause the accumulation of misfolded protein aggregates. In addition, aggregation of α-synuclein and deficiency in PD-related genes can impair neuronal mitophagy and mitochondrial homeostasis. It is crucial to find out the key factors and their roles involved in the pathogenesis of different form of PD. Further studies aiming at modulating the process of autophagy accurately and individually may provide novel therapeutic strategies for this widespread disease.

## Author Contributions

FG, JY, DW, CL, YF, and HW wrote the manuscript. WH and JZ edited the manuscript.

## Conflict of Interest Statement

The authors declare that the research was conducted in the absence of any commercial or financial relationships that could be construed as a potential conflict of interest.
